# Basic stimulus processing alterations from top-down cognitive control in depression drive independent temporal components of multi-echo naturalistic fMRI data

**DOI:** 10.1038/s41398-025-03386-4

**Published:** 2025-05-16

**Authors:** Tengfei Feng, Arnim Johannes Gaebler, Micha Keller, Jana Zweerings, Huanjie Li, Fengyu Cong, Klaus Mathiak

**Affiliations:** 1 Department of Psychiatry, Psychotherapy and Psychosomatics, Faculty of Medicine, RWTH Aachen University, Aachen, Germany; 2 School of Biomedical Engineering, Faculty of Medicine, Dalian University of Technology, Dalian, China; 3 JARA-Translational Brain Medicine, RWTH Aachen University, Aachen, Germany; 4 Institute of Neurophysiology, Faculty of Medicine, RWTH Aachen University, Aachen, Germany; 5 Faculty of Information Technology, University of Jyvaskyla, Jyvaskyla, Finland; 6 Key Laboratory of Social Computing and Cognitive Intelligence (Dalian University of Technology), Ministry of Education, Dalian, China

**Keywords:** Depression, Prognostic markers, Neuroscience

## Abstract

Perceptual changes in major depressive disorder (MDD) may extend beyond emotional content and include the processing of basic stimulus features. These alterations may ultimately contribute to perceptual bias and anhedonia. To characterize blood oxygen level-dependent (BOLD) signal of perceptual processing, we investigated temporally independent fMRI signal components related to naturalistic stimulus processing in 39 patients with MDD and 36 healthy subjects. Leveraging the capability of multi-echo data to detect BOLD activity changes, we extracted physiologically meaningful group temporal components. For each component that exhibited a significant correlation with the movie content, we localized its underlying brain network and assessed MDD-associated alterations. Two components exhibited significant group differences; one was associated with auditory features (sound pressure level) and one with visual features (temporal contrast of intensity). Notably, these deficits in MDD localized primarily to higher-order processing areas, such as the dorsal prefrontal cortex and insula, rather than primary sensory cortices. For the visual feature component, additional group differences emerged in non-visual primary sensory cortices (auditory and somatosensory) as well as major hubs of the motor system. Our findings support the hypothesis that basic sensory processing deficits represent an inherent feature of MDD which may contribute to anhedonia and negative perceptual bias. These deficits are primarily confined to higher-order processing units, as well as cross-modal primary sensory cortices indicating predominant dysfunction of top-down control and multisensory integration. Therapeutic effects of interventions targeting the prefrontal cortex may be partially mediated by restoring prefrontal control not only over emotional but also sensory processing hubs.

## Introduction

Major depressive disorder (MDD) is a highly prevalent mental disorder recognized as one of the leading global causes of disability [1]. On a cognitive level, patients suffer from negative views of themselves, their environment, and their future (Beck’s cognitive triad) [2]. Such negative cognitive distortions may be partially driven by a perceptual bias toward mood-congruent information [3]. For instance, a negative bias in facial emotion recognition emerges during depressive episodes which can be reversed with antidepressant treatment [4]. In addition, a growing body of evidence suggests deficits at the level of early sensory information processing including diminished visual contrast gain [5], reduced auditory pitch identification [6] as well as impaired auditory change detection [7, 8]. Such low-level sensory processing deficits may ultimately contribute to the depressive-core symptom of anhedonia by hindering the generation of enjoyable (hedonic) experiences [9]. Despite the identification of basic sensory deficits, the precise origin and nature of such impairments remains unclear. It is debated whether these arise from dysfunctions within the primary sensory cortices [10], altered sensory processing at lower levels, suggesting bottom-up deficits [5] or alteration in higher-order processing areas, indicating top-down deficits [8]. Moreover, conventional experimental approaches to studying perceptual deficits in MDD may not fully capture the complexities of real-world information processing by the brain processes, as they often lack the contextual depth and dynamic nature characteristic for real-life situations [11]. To address this gap, naturalistic paradigms, such as videos and music, which encompass dynamic stimuli encountered in daily life, present a more realistic approximation of real-world stimuli [11, 12]. Thus, the investigation of alterations in responses to complex naturalistic settings in MDD may provide new insights about perceptual alterations in the disorder [13, 14].

The analysis of naturalistic data poses a critical challenge and necessitates the integration of advanced analytical methods that extend beyond traditional general linear model (GLM) approaches typically employed in task-designed experiments [11]. Inter-subject correlation (ISC), an effective method measuring response correlation across participants, has emerged as a powerful tool for extracting shared responses to naturalistic stimuli [15]. ISC has demonstrated successful applications across diverse tasks such as music listening [16], story listening [17], and film viewing [18]. In affective disorders, ISC has shown reduced synchronization in patients with MDD during exposure to both negative and positive video clips [13]. However, ISC is limited in distinguishing responses to different content within the stimuli (e.g., movie, music) [15, 19]. To overcome these limitations, independent component analysis (ICA), a blind source separation algorithm, has been widely applied for extracting hidden networks within fMRI data [20–22]. Tensor-ICA, specifically, has been instrumental in revealing distinct independent functional networks from multi-subject fMRI data, shedding light on changes based on the associated temporal fluctuations of the BOLD signal in MDD [13]. To date, there is limited research investigating the functional changes underlying responses to naturalistic stimuli, resulting in a scarcity of findings related to MDD. Notably, temporal ICA holds promise in identifying independent content-related responses and hidden group temporal fluctuations, potentially leading to a better understanding of the neural correlates of MDD.

The identification of independent temporal modes holds promise in uncovering spatially overlapping functional networks, particularly as individual brain regions may contribute to various tasks [23, 24]. Despite this potential, temporal ICA faces challenges as it is less robust than spatial ICA due to computational complexities and limited temporal resolution [24]. To obtain temporally-independent functional modes in group data, previous investigation used temporal ICA on the remaining associated time courses of group-wise spatial ICA after artifact identification [24, 25]. Recently, multi-echo (ME) echo-planar imaging (EPI) has gained popularity, leveraging its ability to distinguish between BOLD signal changes and non-BOLD noise through echo-time dependent analysis [26, 27]. Our study validated the feasibility of tensor-ICA to characterize the distribution of neural and non-neural components across TEs [28]. Thus, leveraging true BOLD changes identified from ME-EPI through spatial and temporal ICA, we aimed to identify physiologically meaningful alterations of the processing of naturalistic stimuli in patients with MDD. Specifically, we expected to identify deficits related to basic sensory processing. Given the pivotal role of the prefrontal cortex in the pathophysiology of MDD and our own findings on auditory change detection in MDD [8], we hypothesized that these deficits predominantly originate from higher levels of the sensory processing hierarchy, such as the prefrontal cortex, suggesting a top-down deficit.

## Methods and materials

### Participants and experiment

Thirty-nine individuals diagnosed with MDD (18 females, 35.0 ± 12.9 years old) and 36 healthy controls (15 females, 33.2 ± 13.2 years old), matched for age (t(73) = −0,56, p = 0.56) and gender ratio (chi2 = 0.15, p = 0.70), finished the naturalistic scan and were included in this investigation. This study initially recruited 42 MDD and 39 healthy controls under the guidance of the preregistration (NCT03183947 at clinicaltrials.gov) aiming to detect large effect size. Three participants for each group who did not finish the scanning were excluded from further analysis. All participants were required to have sufficient proficiency in German, normal or corrected-to-normal vision, and be right-handed. Exclusion criteria included MRI contraindications, history of traumatic brain injury, neurological disorders, severe suicidal thoughts, or an inability to provide informed consent. Additionally, healthy controls were excluded if they had a history of psychiatric conditions, as determined by the screening section of the German version of the Structured Clinical Interview for DSM-IV-TR (SCID-I) [29]. Patients diagnosed with acute MDD were included based on formal psychiatric assessment. Comorbid conditions were permitted as long as MDD remained the primary diagnosis. A licensed psychologist verified all diagnoses using DSM-IV-TR criteria [29]. On average, participants with MDD had experienced 4.5 depressive episodes ( ± 5.7) and had been living with the disorder for 8.4 years ( ± 8.0). None of the MDD group fulfilled criteria for psychotic depression. Those taking medication maintained a stable regimen for at least one week prior to and throughout the study. For the MDD group, the mean antidepressant medications received were 187% ± 147% (percentage of the defined daily dose, DDD). Seventeen out of the MDD group taking 247% ± 94% SSRI medication. The BDI-II scores (Beck Depression Inventory, Second Edition [30]) were 3.78 ± 4.24 and 24.44 ± 12.70 for the healthy and MDD group respectively (t = −9.29, p < 0.001). The HAMD scales (Hamilton Rating Scale for Depression [31]) for the MDD group were 15.97 ± 7.57. All the participants were recruited from the same participant pool as utilized in a previous study [32]. More detailed demographic information about the participants can be found in this earlier study. The research adhered to the principles outlined in the Declaration of Helsinki and received approval from the Local Ethics Committee of RWTH Aachen University Hospital (EK 216/11). All participants provided written informed consent after receiving a thorough explanation of the study and its procedures.

Participants watched a 20-minute excerpt from the German film “Lola rennt” (English: “Run Lola Run”) from the 10:20 to 30:20-minute mark (X-Filme Creative Pool, Germany, 1998). This segment was selected for its self-contained narrative and dynamics of a fast-paced storyline [33]. The video was presented using a projector system equipped with reflecting mirrors (Psychology Software Tools; Sharpsburg, PA, United States). Participants received the audio stimulation through earplugs from Nordic Neurolab (Bergen, Norway), with individually adjusted sound levels.

Data was collected using a Siemens 3 Tesla MRI scanner (MAGNETOM Prisma Fit, Simens Medical Systems, Erlangen, Germany) equipped with a 20-channel head coil. To acquire ME-data at a high repetition rate, multiband ME-EPI technique in conjunction with parallel imaging (PI) was applied to collect T2*-weighted images. Four contrast images (echo time = 13, 28, 43, and 57 ms) were acquired using ME-EPI which covers the most optimal TEs for different areas. Such a collection strategy has been shown to reduce imaging artifacts while conserving BOLD sensitivity [34] and verified the ability to distinguish BOLD and non-BOLD change [28]. iPAT factor for PI were set to 2 and TR was 1 s for a high temporal resolution. Matrix size was 64×64 acquired with 36 transverse slices using threefold multiband acceleration. The flip angle was set to 67 degrees approximating Ernst’s angle. In total, 1220 repetitions for every subject were collected during movie watching (10 seconds before and after the movie were recorded). The sequence parameters for T1-weighted 1mm-isotropic anatomical images are TR = 2000 ms, TE = 3.03 ms, T1 = 900, matrix = 256×256, and 176 sagittal partitions. All participants were instructed to minimize movement during scanning.

### Extraction of shared group temporal components

**Fig. 1:**
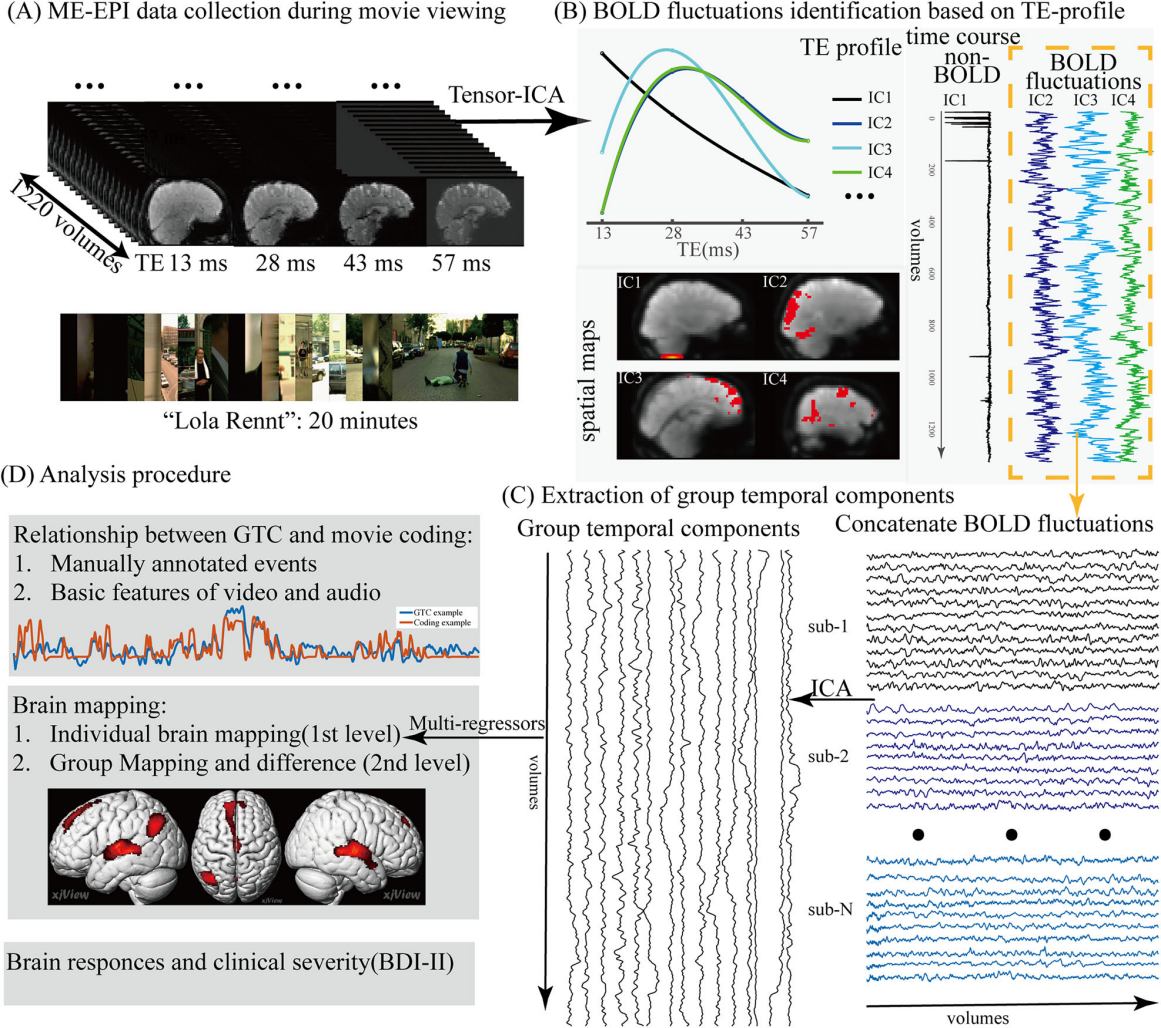
Schematic overview of this study. **A** Multi-echo EPI data were collected during a 20-minute movie clip viewing session (“Lola Rennt”). **B** Individual BOLD fluctuations identification based on three-way decomposition of the ME-EPI data. The data were first decomposed into spatial, temporal, and TE domains. Components exhibiting BOLD patterns (bell-shaped) across TEs were identified as BOLD changes. **C** Extraction of group temporal components using ICA. The BOLD fluctuations from all subjects were concatenated, forming a matrix of all BOLD time series. ICA was then utilized to decompose the group time series and derive the underlying shared responses at the group level. **D** Further analyses contained in the context of the group temporal components in this study.

Two steps were employed to extract group temporal responses (Fig. 1B, C). Firstly, individual BOLD fluctuations were identified based on the TE profile from ME-EPI data decomposed with tensor-ICA. Secondly, group-level underlying responses were derived by decomposing the concatenated BOLD time series across subjects with ICA. Before the decomposition process, motion correction was performed by realigning all echo data using motion parameters estimated from the first echo (TE = 13 ms). Additionally, the data were smoothed with a 4 mm full width at half-maximum (FWHM) Gaussian kernel. Subsequently, tensor ICA, implemented in the Multivariate Exploratory Linear Decomposition into Independent Components (MELODIC) tool within the FSL toolbox (fsl.fmrib.ox.ac.uk/fsl), was employed to decompose the ME-data into spatial, temporal, and TE domains. The number of components was determined automatically using the minimum description length (MDL) criteria [35]. Given that BOLD changes exhibit distinct characteristics from non-BOLD changes, which typically display an exponential decay pattern in the TE domain, the identification of BOLD time courses involved assessing whether they showed peaks between 20 and 50 ms across TEs. The peaks were fitted by quadratic polynomial curves and only these components were further considered. Subsequently, the time sequence of identified BOLD components underwent moving-average filters with a window size of 5 before being fed into the temporal ICA on the group level. The group ICA utilized the package ICASSO (https://research.ics.aalto.fi/ica/icasso/) [36] to improve the reliability of components by clustering after 20 runs. The number of components was set as suggested by MDL criteria.

### Correlations between shared temporal components and movie coding

To investigate the relationship between shared temporal components within the group and specific stimuli presented in the movie, a comprehensive annotation process was undertaken. Various stimuli, encompassing language presence, facial expressions (categorized as positive, neutral, or negative), music presence, person presence, and more, were manually annotated frame-by-frame by three coders [33]. Additionally, basic features extracted from the video and audio were taken into account, following the approach outlined in a previous study [33, 37]. These features included luminance (mean intensity across pixels of frames), temporal contrast (mean absolute temporal derivative of intensity change), local contrast (mean absolute difference between one pixel and its surrounding pixels), sound pressure level (SPL) in dB level, and sound envelope (the squared magnitude of the Hilbert transform of SPL). Further, standard deviation (std) and mean squared (mSq) values of luminance, temporal contrast, and local contrast were computed. Subsequently, these annotated stimuli were convolved with the hemodynamic response function to model the task-related responses. The Pearson correlation coefficients (cc) were then calculated between all group-shared responses and all annotated stimuli. To control for multiple comparisons, p-values were corrected according to false discovery rate (FDR) below 0.05. This analysis targeted the associations between the shared neural responses and specific features or cues present in the naturalistic movie-watching paradigm.

### Brain mappings for derived group temporal components

To localize the identified group-temporal components in the brain, GLM analysis was conducted in SPM12 (Wellcome Trust Center for Neuroimaging, London, UK). Here, the fMRI data were first preprocessed involving the following steps. Firstly, the ME data were combined, assigning approximated weights of [1 2 2 2] to the four echoes for optimized BOLD sensitivity [34, 38]. To mitigate saturation effects, the first 10 volumes of each run were discarded. Subsequently, standard preprocessing procedures were sequentially executed, including realignment, co-registration, normalization, and smoothing with an 8-mm FWHM Gaussian kernel.

For the first level GLM analysis, the respective components derived from the group temporal ICA were utilized simultaneously as regressors. The corresponding beta maps, representing responses to each of the group components, subsequently entered a second-level analysis. First, a one-sample t-test including all subjects assessed brain networks associated with each temporal component on the whole group level, considering both positive and negative sides since ICA components are arbitrary. Subsequently, Pearson correlation coefficients (cc) were calculated between all group-shared responses and all annotated stimuli. To control for multiple comparisons, p-values were corrected according to a false discovery rate (FDR) below 0.05.

### Brain responses and clinical measures

Group differences in depressed patients may relate to trait or state variables. To evaluate the relationship between brain responses and symptom severity, medication, or other clinical measures, we extracted the beta values of each subject at the cluster peaks with significant group differences. Subsequently, Pearson correlation coefficients were calculated between the extracted beta values and the BDI-II score, HAMD score, dosage of psychopharmacological medication, dosage of SSRIs, and duration of illness. Significant associations indicate an association of movie-related brain activity with the clinical state.

## Results

During the identification of BOLD fluctuations from individual participants, an average of 13.2 ± 4.3 components were labeled as BOLD changes and subsequently considered for group ICA. The number of components for group ICA was set to 15. Group ICA was run 20 times, and the ICASSO toolbox was used to cluster the derived components from these runs. These components were clustered into 15 groups, each demonstrating high stability with a stability index of 0.85 ± 0.08. Finally, 15 robust components emerged from the group ICA of the individual components, which exhibited a BOLD-like TE profile. Subsequently, temporal ccs between each group temporal component (GTC) and the content series, after hemodynamic convolution, were computed. Significant correlations with absolute values greater than 0.35 were observed in 8 out of the 15 GTCs, with all p-values < 0.05 after FDR correction (Table 1).

**Table 1:** The correlation coefficients between group temporal components (GTCs) and representative movie coding.

GTC#	Exp. Var.	Movie content coding				Auditory and visual stimulus features			
		Language	Face	Music	Negative face	SPL	mSq of temporal contrast	std of local contrast	Sound envelope
1	0.08	**0.677**	0.215	0.196	0.311	−0.268	−0.226	0.259	−0.211
2	0.11	0.245	−0.050	0.070	−0.011	0.129	0.192	−0.048	0.134
3	0.11	0.186	0.026	−0.121	−0.121	0.210	0.099	**0.488**	**0.365**
4	0.05	−0.060	−0.167	−0.291	0.043	**0.403**	−0.038	−0.039	0.234
5	0.06	0.174	0.128	0.037	0.048	−0.058	0.162	0.045	0.011
6	0.05	0.020	−0.080	0.058	0.139	−0.091	0.069	0.013	−0.062
7	0.04	−0.135	0.052	−0.061	−0.019	−0.073	0.191	−0.124	−0.076
8	0.04	−0.223	**−0.480**	−0.100	−0.274	0.053	−0.082	−0.129	0.138
9	0.04	−0.064	−0.088	−0.101	0.070	0.124	0.095	−0.132	0.067
10	0.09	−0.078	−0.227	**−0.359**	−0.261	0.143	0.046	0.146	−0.053
11	0.06	0.118	0.166	0.047	0.107	−0.049	−0.120	0.008	−0.069
12	0.06	−0.118	0.197	−0.041	−0.127	0.129	**0.505**	−0.047	**0.361**
13	0.06	0.014	−0.063	−0.250	−0.264	0.256	0.136	−0.120	0.275
14	0.12	0.188	0.202	−0.019	**0.390**	−0.130	−0.198	0.010	−0.243
15	0.06	−0.100	−0.120	−0.016	0.015	0.103	**0.358**	0.002	0.233

Significant correlations exhibiting absolute values larger than 0.35 and FDR-corrected p-values below 0.05 are set in bold.

*Exp. var*. explained variance; *SPL* sound pressure level; *mSq* mean square; *std* standard deviation.

### Stimulus-related temporal components

**Fig. 2:**
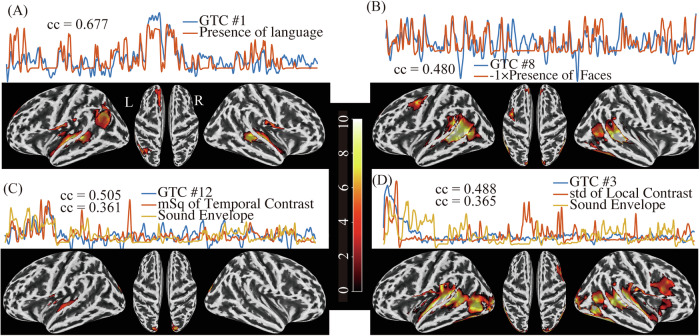
Representative group components (GTC) with significant stimulus correlations and their underlying brain maps (t-value). **A** GTC #1 (blue line: normalized time course over the 20 min video) correlated significantly (cc = 0.677, p < 0.001) with the manually annotated presence of language after convolution with hemodynamic response function (red line). The statistical map projected on the brain surface reflects a linear regression with this GTC. **B** GTC #8 correlated with the presence of faces (cc = 0.480, p < 0.001). **C** and **D** Two GTCs yielded significant ccs with basic stimulus features extracted from the video (red line) and audio signal (yellow line; see text for details). (GTC: group temporal components; cc: correlation coefficient).

Among the eight movie-related GTCs, four of them correlated with manually-coded movie content (presence of spoken language, faces, or music) whereas the other half was associated with basic auditory and visual stimulus features (see Fig. 2, for representative components). GTC #1 exhibited a particularly robust correlation of 0.67 (descriptive p < 0.001) with the presence of language coded in the movie (Fig. 2A). Regression of this GTC in each participant led to an associated group activation map in the second level analysis: activation to language content emerged at bilateral temporal (superior temporal, Heschl’s gyrus, Rolandic operculum), left inferior parietal (angular gyrus), and parts of the bilateral medial and superior frontal lobule. Similarly, GTC #8 exhibited a correlation coefficient of 0.48 (descriptive p < 0.001) with the presence of faces in the movie scenes (Fig. 2B). The associated brain areas were primarily located in the bilateral temporal (middle temporal, fusiform, and superior temporal gyrus), a left pre-motor, and occipital lateral areas. In addition to these manually annotated stimuli, some components demonstrated high correlations with extracted auditory or visual features of the movie. For instance, GTC #12 displayed a significant correlation of 0.505 with the feature of luminance changes and correlation of 0.361 with the sound envelope (Fig. 2C). Further, GTC #3 was correlated with the std of local contrast (cc = 0.488, p < 0.001) and the sound envelope (cc = 0.365, p < 0.001; Fig. 2D). The brain maps of these two components highlighted contributions of super-temporal and lateral occipital areas, corresponding to visual and auditory processing regions, respectively.

### Altered responses to naturalistic stimuli in MDD

**Fig. 3:**
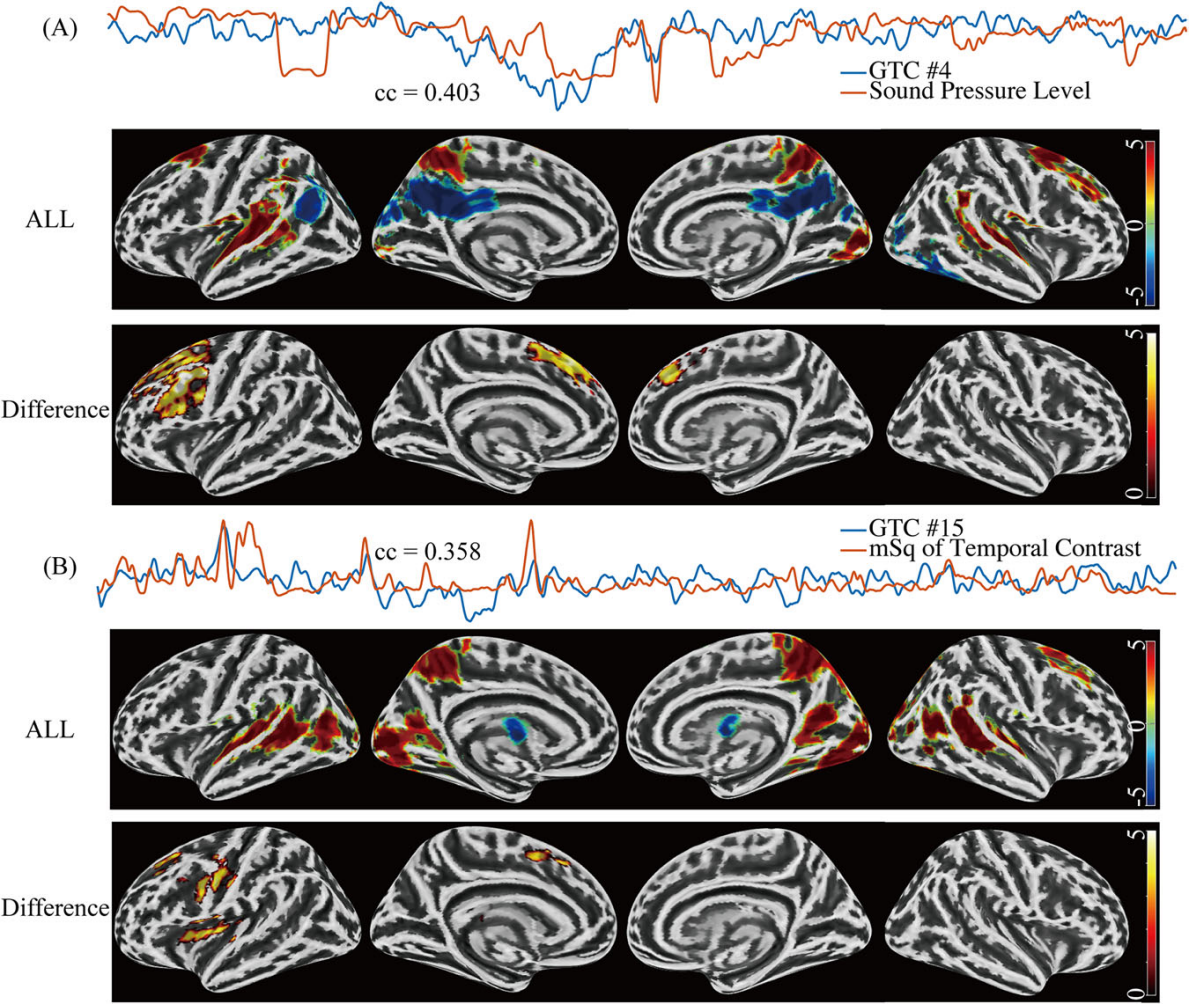
Group differences emerged in the mapping of two shared responses (p < 0.05 FWE-corrected). Both these GTCs were characterized by sensory processing. **A** GTC #4 correlated with sound pressure level and revealed group differences in the left dorsal prefrontal dominant area between HC and MDD groups. **B** GTC #15 correlated with a visual stimulus feature and with group differences in four left hemispheric clusters around the SFG, pre- and postcentral gyrus, insula, and thalamus.

Among the 15 GTCs, two revealed significant differences between the MDD and the control group in the brain mapping (p < 0.05 FWE-corrected; Fig. 3). More specifically, GTC #4 exhibited a correlation of 0.403 with the auditory stimulus of SPL (Fig. 3A). The spatial distribution of this component converged on the bilateral temporal (e.g., superior and middle temporal gyrus), parietal (e.g., supramarginal gyrus, calcarine) and frontal (e.g., superior and middle frontal gyrus) lobules and deactivation in precuneus. Within this response, we observed a specific cluster indicating significant group differences in the left dorsal prefrontal cortex (cluster #1a). Furthermore, GTC #15 displayed a correlation of 0.358 with the visual stimulus feature of mSq of temporal contrast (Fig. 3B), and our analysis revealed 4 distinct left-hemispheric clusters showcasing group differences within regions encompassing the left SFG (cluster #2a), pre- and postcentral gyrus (cluster #2b), insula (cluster #2c), and thalamus (cluster #2 d; Table 2). The functional annotations of the thalamic cluster retrieved from the Neurosynth platform (neurosynth.org) included the terms “finger” (z-score = 7.53, posterior probability = 0.79) and “motor” (z-score = 6.67, posterior probability = 0.67), thus suggesting its assignment to the motor thalamus in line with the motor-sensory cluster. The other components showed no group difference at the corrected mapping threshold.

**Table 2:** Clusters with significant difference between HC and MDD.

Cluster #	Brain regions	Peaks’ MNI coordinates			T	k_E_
		x	y	z		
Auditory stimulation-related group difference						
1a	Left MFG, SFG, Precentral, SMA, IFG_triang_, IFG_operc_, Left and right SFG_medial_	−46	12	44	6.34	4255
Visual stimulation-related group difference						
2a	Left SFG, SMA	−14	24	46	4.59	570
2b	Left Precentral, Postcentral	−54	0	32	4.56	489
2c	Left Insula, Putamen, Heschl’s gyrus, Rolandic operculum	−40	2	6	4.43	473
2d	Left Thalamus	−14	−24	10	4.93	312

The regions were labelled according to the automatic anatomical labeling atlas (AAL3). Cluster-level threshold according to p_FWE_ < 0.05 after voxel-level threshold p < 0.001.

### Brain behavior relationship

**Fig. 4:**
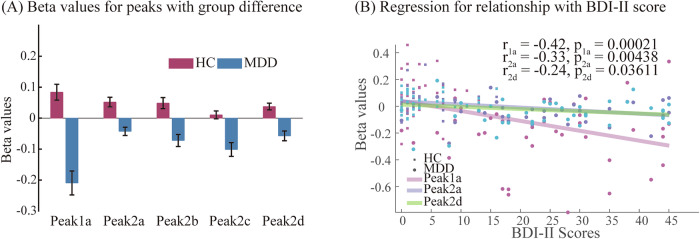
Brain alterations and relationship with clinical symptom severity. **A** Beta values from clusters with significant group differences (error bar is the standard error of the mean). **B** Significant correlations between peaks from group differences and depression severity (BDI-II score).

From the five cluster peaks reflecting group differences, beta values were extracted to examine the relationship between altered responses and symptom scores (Fig. 4). According to their MNI coordinates in the AAL atlas, these five peaks were located in the left MFG, SFG, precentral, insula, and thalamus. The values of the five clusters for the HC group were 0.084 ± 0.154, 0.052 ± 0.093, 0.048 ± 0.107, 0.011 ± 0.076, and 0.038 ± 0.066. For the MDD group, these values were −0.209 ± 0.242, −0.043 ± 0.084, −0.072 ± 0.123, −0.101 ± 0.138, and −0.057 ± 0.099. Three peaks displayed significant correlations with the symptom severity score, as measured by the BDI-II. The MFG peak exhibited a negative correlation of −0.41 with BDI-II score (p < 0.001). Additionally, the peaks in the SFG and thalamus demonstrated significant correlations with BDI-II scores (−0.33 and −0.24; p < 0.05). No significant correlation emerged between the extracted beta values and other tested clinical assessments (HAMD; p > 0.15), medication (total and SSRI DDD; all p > 0.3) or duration of illness (p > 0.4).

## Discussion

The present study investigated the neural correlates of perceptual changes in patients with MDD by analyzing group temporal components related to the processing of naturalistic stimuli. Utilizing ME-EPI data which can distinguish BOLD changes from non-BOLD noise, we extracted physiologically meaningful group temporal components (GTCs). Several of these GTCs showed significant correlations with the annotated movie content. Notably, at the specified threshold, alterations in patients only emerged for components associated with basic auditory or visual features of the movie. Specifically, differences were related to the sound pressure level and the mean square of the temporal luminance contrast. The observed differences were not primarily located in the associated primary or secondary auditory or visual cortices but in higher-level processing areas, including the dorsal prefrontal cortex and the insula. For the visual feature component, additional group differences emerged in primary sensory cortices of non-visual domains. These included the primary auditory and somatosensory cortices as well as nodes belonging to the motor system such as the primary motor cortex and motor thalamus.

Converging evidence suggests basic sensory processing deficits as an inherent feature of MDD potentially contributing to one of its defining symptoms, anhedonia [9]. Examples from the literature include diminished visual contrast gain [5], enhanced visual motion perception [39], reduced auditory pitch identification [6], auditory sensory gating [40], as well as impaired auditory change detection [8]. However, there is considerable debate on the spatial origin of the respective processing deficits in the sensory hierarchy and the relative contribution of bottom-up and top-down deficits. In the auditory domain, our current findings align with our previous studies on deviance detection (mismatch responses) suggesting a predominant deficit of higher-level prefrontal and parietal processing nodes. However, there was also evidence for a minor contribution of the auditory cortex [8]. In contrast, patients with schizophrenia exhibited mismatch deficits already in early subcortical processing nodes including the inferior colliculus and thalamus thereby distinguishing them from patients with major depressive disorder [41]. For the visual domain, findings of lower retinal contrast gain detected by pattern electroretinogram (PERG) may indicate very early processing deficits located at the level of the retina [5]. However, a recent behavioral study revealed that while brightness induction was intact, contrast suppression was significantly lower in patients with MDD than in controls suggesting normal retinal but altered cortical processing [10]. In a similar manner, a previous study detected deficient top-down control of the visual cortex by frontoparietal networks in MDD, leading to abnormal filtering of irrelevant visual information [42]. In the present study, for the temporal component related to the visual stimulus feature, no group differences emerged in primary visual brain areas. Again, alterations in MDD involved the prefrontal cortex, but also brain hubs involved in processing non-visual sensory information (auditory and somatosensory cortex) as well as parts of the motor system (primary motor cortex and motor thalamus), suggesting dysfunctional cross-modal interactions and multisensory integration.

Similarly, previous studies have suggested deficits in multisensory integration for audiovisual emotional information [43, 44]. As the present study indicates, MDD-associated deficits in cross-modal interactions may also be detected in the context of processing non-emotional information suggesting a more fundamental dysfunction of multisensory integration. The predominant deficit of higher-order processing units – particularly the prefrontal cortex - prompts a primary top-down deficit in MDD and confirms its central role in the pathophysiology of MDD. In this context, previous studies identified hypo-activation of the dorsolateral prefrontal cortex (DLPFC) [45], deficits in downregulating amygdala responses during presentation of negative stimuli leading to a failure to regulate negative mood [46, 47] and expansion of the frontostriatal salience network [48]. Additional deficits in the top-down control of sensory as well as reward processing circuits mediated by the prefrontal cortex may further contribute to anhedonia. Interestingly, the prefrontal activation deficit observed in this study was confined to the left hemisphere. Such a left-lateralized pattern is also supported by a substantial number of studies [49] including our own findings on auditory mismatch processing [8]. Accordingly, the prefrontal cortex is the most common target in repetitive transcranial magnetic stimulation (rTMS) treatment protocols for MDD which typically apply high-frequency (activating) stimulation of the left DLPFC [50] or low-frequency (inhibitory) stimulation of the right DLPFC [51]. Hence, therapeutic effects might be partially driven by restoring the control of the left DLPFC over brain hubs involved in emotional, reward, and sensory processing.

The loudness dependence of the auditory evoked potential (LDAEP) is considered a marker for auditory cortex responsiveness and central serotonergic function. Indeed, stronger LDAEP has been consistently documented in patients with MDD and stronger baseline LDAEP correlated with greater clinical response to SSRIs in depression and anxiety disorders [52]. Moreover, recent studies suggest a close association between LDAEP and brain activity in affective disorders, supporting its role as a non-invasive biomarker of serotonergic modulation [53]. The IC-maps and difference maps were compared with 5HT-receptor maps [54]. However, we did not find any significant similarity between the maps in this study and the five 5HT-receptor distributions. In our study, we found altered fMRI responses to sound pressure levels in MDD, particularly in higher-order auditory and multisensory integration areas, including the dorsal PFC and insula. These regions are involved in top-down modulation of sensory input which may suggest that serotonergic dysfunction may extend beyond primary auditory processing and impact higher-order sensory integration as well. Besides, no significant correlations were observed between the altered neural response and the SSRI or SNRI medication group. Future research could explore pharmacological manipulations and multimodal imaging approaches to further investigate these relationships.

Apart from these findings, further works may address the limitations of this work. The natural viewing condition used in this study did not include behavioral measures, thus, no joint analysis of brain functional deficits and behavioral perceptual deficits was conducted. Further studies integrating such measures may shed light on the relationship between functional deficits and different types of perceptual deficits [8]. Additionally, considering the limited sample size, only weak correlations of component amplitudes with symptomatology were observed. In this study, we aimed to investigate perceptual alterations using temporal ICA components. Both ISC and ICA share a common assumption that participants exposed to the same naturalistic stimuli will exhibit similar brain response patterns [21, 49]. While ISC relies on correlation-based constraints, temporal or spatial ICA estimate distinct independent brain fluctuation patterns or spatial patterns, respectively [17, 19, 24]. Each method has its own strengths and limitations. Additionally, spatial ROI seeds-based functional connectivity (FC) could be investigated, and especially dynamic FC may be reasonable and differentiate hidden responses within the long scanning. Consequently, employing different methods may provide complementary insights into brain responses during naturalistic stimuli processing.

In conclusion, the present study demonstrates that extracting physiologically meaningful independent temporal components from ME-EPI data offers a valuable approach to delineate perceptual alterations in mental disorders such as MDD. Our findings add to a growing body of research suggesting basic sensory processing deficits as an inherent feature of MDD, which may ultimately contribute to anhedonia and cognitive bias. These deficits primarily localize to higher-order processing units such as the prefrontal cortex rather than primary sensory cortices, thus confirming its pivotal role in the pathophysiology of MDD. Future studies should address how therapeutic interventions targeting the prefrontal cortex (such as rTMS) affect the brain network changes identified in this study as well as their contribution to the overall treatment response.

## Data Availability

The scripts for reproducing the results are available on request the corresponding author and core scripts for extracting components were shared on Github (https://github.com/TengfeiFeng/MDD_Temporal_Response_to_NS). The data is available upon request if not limited by concerns by the ethical commission.
